# The Effects of Chlormadinone Acetate on Lower Urinary Tract Symptoms and Erectile Functions of Patients with Benign Prostatic Hyperplasia: A Prospective Multicenter Clinical Study

**DOI:** 10.1155/2013/584678

**Published:** 2013-05-16

**Authors:** Kiyohide Fujimoto, Yoshihiko Hirao, Yasuo Ohashi, Yasuhiro Shibata, Kohzo Fuji, Hidenori Tsuji, Katsuhito Miyazawa, Mikinobu Ohtani, Ryoji Furuya, Eitetsu Boku

**Affiliations:** ^1^Department of Urology, Nara Medical University, 840 Shijo-cho Kashihara, Nara 634-8522, Japan; ^2^Department of Biostatistics, University of Tokyo, Tokyo 113-0033, Japan; ^3^Department of Urology, Gunma University Graduate School of Medicine, Maebashi 371-8511, Japan; ^4^Department of Urology, Showa University School of Medicine, Tokyo 142-8555, Japan; ^5^Department of Urology, Kinki University Faculty of Medicine, Osaka-Sayama 589-8511, Japan; ^6^Department of Urogenital Surgery, Kanazawa Medical University, Kanazawa 920-0293, Japan; ^7^Department of Urology, Ibaraki Prefectural Central Hospital and Cancer Center, Kasama 309-1793, Japan; ^8^Department of Urology, Furuya Hospital, Kitami 090-0065, Japan; ^9^Boku Clinic of Urology and Nephrology, Habikino 583-0856, Japan

## Abstract

*Purpose*. To evaluate the effects of chlormadinone acetate (CMA), progesterone-derived antiandrogen, on lower urinary tract symptoms (LUTS) and erectile functions of benign prostatic hyperplasia (BPH). *Methods.* A multicenter, single-cohort prospective study was conducted. A total of 114 patients received CMA for 16 weeks. The endpoints were changes in International Prostate Symptom Scores (IPSS), IPSS-QOL, International Index of Erectile Function-5, *Q*_max_ prostate volume, and residual urine volume. *Results.* Significant improvements were observed in IPSS from week 8 to week 48 (32 weeks after treatment). IPSS-QOL improvements were also significant from week 8 to week 48. *Q*_max_ increased to a maximum at Week 16 and remained elevated throughout the study. Moreover, a decrease of 25% in prostate volume was observed at Week 16. IPSS, QOL, and Qmax changes during the study were not different between the previously treated and untreated patients. IPSS storage subscore changes differed between the age groups. Few severe adverse reactions were observed, except for erectile dysfunction. *Conclusions.* CMA rapidly and significantly reduced prostate volume and improved voiding and storage symptoms and QOL. Our results suggest that CMA is safe and beneficial, especially for elderly patients with LUTS associated with BPH.

## 1. Introduction

Cases of glandular hyperplasia and those of mixture type with stromal hyperplasia constitute approximately 90% of benign prostatic hyperplasia (BPH) cases; therefore, antiandrogens are very likely effective in most patients with BPH reducing prostate volume, relieving mechanical obstructions at the prostatic urethra, and improving urinary flow. 

Meanwhile, 5-alpha reductase inhibitors (5-ARIs), which inhibit the conversion of testosterone to dihydrotestosterone, have been approved for treating BPH. Because adverse effects on sexual function are less frequently encountered with 5-ARI treatment than with antiandrogen treatment, the 2012 Guidelines of the European Association of Urology recommend 5-ARIs, including dutasteride, as a first-line treatment for BPH in patients with large prostate volumes of 40 mL or more. Conversely, for patients with small prostate volumes of less than 40 mL, anticholinergic treatment with an *α*1-blocker is first recommended [1]. The Medical Therapy of Prostatic Symptoms (MTOPS) study also concluded that patients with baseline prostate volumes of 31 mL or more showed a high rate of clinical progression of BPH such as exacerbation of lower urinary tract symptoms (LUTS), urinary retention, or requiring surgical treatments [2]. In such cases, allopathy with an *α*1-blocker monotherapy is not always the best approach. Instead, treatment with antiandrogens or 5-ARIs is a reasonable choice to reduce adenoma volume and is strongly recommended in cases where *α*1-blockers are ineffective or their efficacies have been attenuated. 

Chlormadinone acetate (CMA), which is a progesterone-derived antiandrogen, mainly inhibits the uptake of testosterone by epithelial cells and the binding of dihydrotestosterone to androgen receptors within the cell nuclei. CMA reduces both levels of testosterone in blood and tissue, including apoptosis in prostate epithelial cells, thereby causing atrophy of adenomas in patients with BPH [3]. 

Previously, we conducted a multicenter, single-cohort prospective study in patients with BPH to investigate the changes in serum prostate-specific antigen (PSA) and testosterone levels and reported that CMA treatment decreased the serum PSA levels by approximately 50% and the testosterone levels by approximately 90% [4]. Herein, we report the changes in the secondary efficacy endpoints of our previous study related to LUTS and sexual function. 

## 2. Methods

### 2.1. Patients and Data Selection

This was a multicenter, single-cohort prospective study. The institutional review board at each study center approved the study design and protocol, and the study was conducted in accordance with the Helsinki Declaration. All patients provided written consent prior to enrollment. The Central Data Center of the Japan Clinical Research Support Unit was responsible for central patient enrollment, data management, and study monitoring. To be included in this study, patients had to meet all of the following criteria: age of 50 years or older; untreated BPH patients meeting the following criteria or BPH patients who were being treated with an *α*1-blocker or anticholinergic agent for more than 1 month and were not planning to change the dosage during this study: baseline PSA values of 10 ng/mL or less; a maximal urinary flow rate (*Q*_max⁡_) of less than 15 mL/s; an estimated prostate gland volume of 20 mL or greater; an International Prostate Symptom Score (IPSS) of 8 or greater; and an IPSS-quality of life (QOL) score of 2 or greater. Patients who met any of the following criteria were excluded from the study: patients with serious hepatic disorders or liver disease; patients with a malignant tumor, including prostate cancer, or with a history of a malignant tumor within 5 years; patients with urethral stenosis interfering with the evaluation of voiding function; patients with a residual urine volume (RU) > 100 mL; patients with a history of transurethral resections of the prostate (TURP), laser therapy, or thermotherapy. Patients were also excluded if they had any of the following medical conditions or a history of the following treatments that are known to affect PSA levels: prostatitis; active urinary tract infection; treatment with any sexual hormones, including antiandrogens and estrogens, within 1 year; treatment with any anti-inflammatory drugs, including Eviprostat (*Chimaphila umbellata* extract, *Populus tremula* extract, *Pulsatilla pratensis* Mill extract, *Equisetum arvense* extract, and refined wheat germ oil; Nippon Shinyaku Co, Ltd., Kyoto), *cernitin* pollen extract or steroid hormones within 3 months; and the presence of an indwelling urethral catheter stent within 4 weeks. 

Patients orally received 25 mg of CMA twice daily or 50 mg of CMA once daily after meals for 16 weeks and were observed for another 32 weeks. Patients who were being treated with an *α*1-blocker or anticholinergic agent continued to receive the same drugs throughout the study without changing the dose. 

### 2.2. Method of Evaluation

The endpoints were the changes in IPSS, IPSS-QOL, *Q*_max⁡_, prostate volume, RU, and the International Index of Erectile Function (IIEF) 5 from baseline to each evaluation point, that is, weeks 8, 16, 24, 32, and 48. Prostate volume was measured at weeks 16, 32, and 48. Clinical laboratory tests, including hematology, clinical chemistry, and urinalysis, were performed at Weeks 0 and 16. All evaluations were also performed when patients discontinued the study for any reason. 

For all analyses, repeated analysis of variance (ANOVA) was used to estimate the least squares (LS) mean and standard error (SE) at each measurement time point. Dunnett's multiple comparison tests were used to compare measurement values at baseline with those at each time point. Interactions of each stratum and time point were tested to explore the possible influences of background factors on the changes over time. *P* values less than 0.05 were interpreted as an indication of statistical significance.

If the total score for IIEF-5 Questions fom 2 to 4 was zero at baseline, the patient was excluded from the IIEF-5 analysis. After a logarithmic transformation, PSA levels, testosterone levels, prostate volume, and RU values were analyzed by repeated-measurement analyses, and the estimates were presented after being transformed by an exponential backtransformation.

## 3. Results

A total of 115 patients were enrolled in this study between March 2007 and March 2009; however, 1 patient did not meet the inclusion criteria. Of the 114 eligible patients, 22 discontinued the CMA treatment with a mean treatment period of 6.0 weeks (0–13 weeks). Four patients discontinued the study because of an adverse event. The baseline characteristics of the patients are presented in Table 1. The mean PSA level was 3.66 ng/mL, and the mean prostate volume was 46.15 mL at baseline. A total of 91 (79.8%) patients were treated with an *α*1-blocker or anticholinergic agent. 

The changes in the IPSS are presented in Table 2. Improvements in the IPSS total score were significant from week 8 to week 48 (*P* < 0.05). The maximum improvement was observed at week 24 (8 weeks after CMA treatment). The mean changes from baseline in IPSS total score were −2.95 at Week 8, −5.49 at Week 24, and −4.34 at Week 48. The IPSS voiding and storage subscores individually showed the same improvement patterns as the total score, whereas little improvement was observed in the nocturia subscore during the CMA treatment. The IPSS-QOL score also improved significantly at week 8 and remained improved after the treatment had ended. 

**Figure 1:**
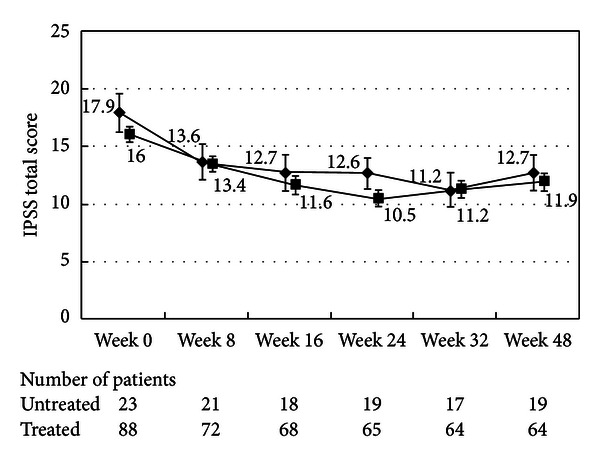
International Prostate Symptom Score total score. The scores in the patients treated with an *α*1-blocker or anticholinergic agent (■) and the untreated patients (◆) are shown. The values are the least squares (LS) mean ± standard error (SE). *P* = 0.1747 (repeated ANOVA).

**Figure 2:**
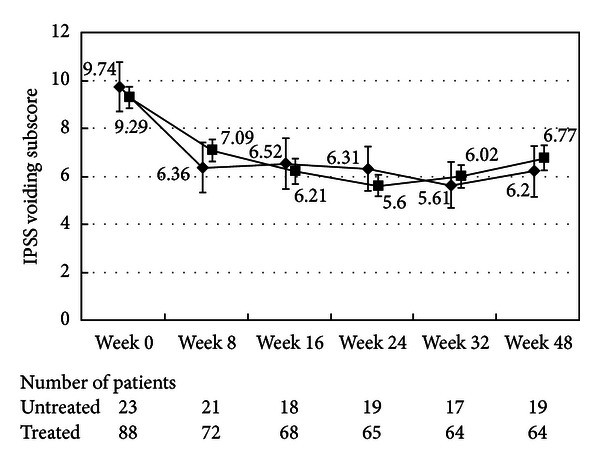
International Prostate Symptom Score voiding subscore (total scores of questions 1, 3, 5, and 6). The scores in the patients treated with an *α*1-blocker or anticholinergic agent (■) and the untreated patients (◆) are shown. The values are LS mean ± SE. *P* = 0.2359 (repeated ANOVA).

**Figure 3:**
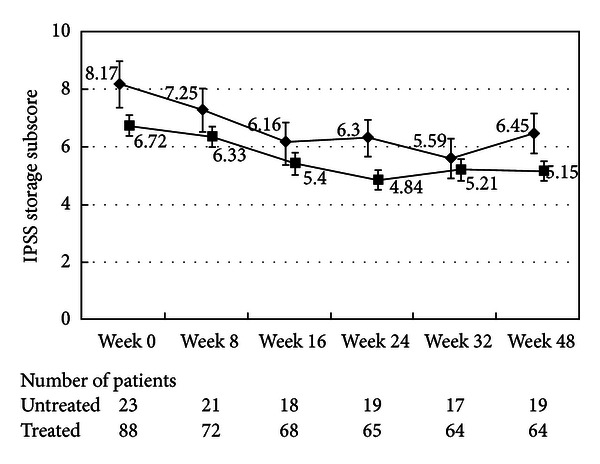
International Prostate Symptom Score storage subscore (total scores of questions 2, 4, and 7). The scores in the patients treated with an *α*1-blocker or anticholinergic agent (■) and the untreated patients (◆) are shown. The values are LS mean ± SE. *P* = 0.0887 (repeated ANOVA).

**Figure 4:**
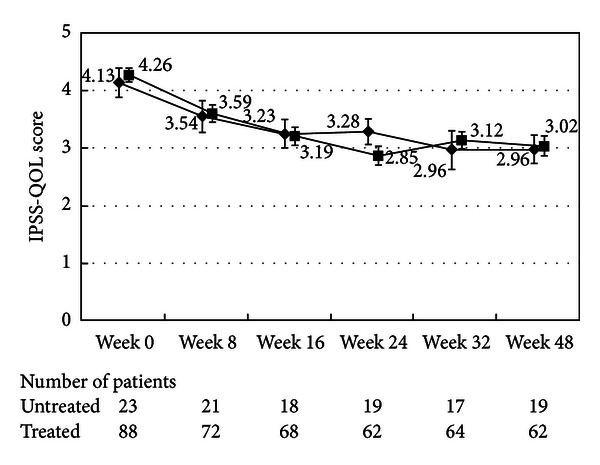
International Prostate Symptom Score quality of life score. The scores in the patients treated with an *α*1-blocker or anticholinergic agent (■) and the untreated patients (◆) are shown. The values are LS mean ± SE. *P* = 0.0554 (repeated ANOVA).

There were no differences in the changes in the total IPSS or IPSS-QOL scores between the patients being treated with an *α*1-blocker or anticholinergic agent and the untreated patients (Figures 1, 2, 3, and 4). Although the changes in total IPSS and voiding subscores were not different between the age groups, the changes in the IPSS storage subscores differed between the age groups (*P* = 0.0290, Figures 7, 8, 9, and 10). 

The mean *Q*_max⁡_ increased to a maximum of 1.96 mL/s at week 16 and remained elevated until week 48. There were no differences in the increases in *Q*_max⁡_ between the patients being treated with an *α*1-blocker or anticholinergic agent and the untreated patients (Figure 6). At Week 16, prostate volume decreased to 75% of that at week 0. 

Neither clinically severe adverse events nor laboratory test abnormalities were observed in this study. The slight elevation of AST and ALT was observed in one patient. Two patients had an increase of urinary frequency. Other low-grade adverse events were anorexia, oral mucositis, hypertension, and depression shown in one patient for each adverse event. 

**Figure 5:**
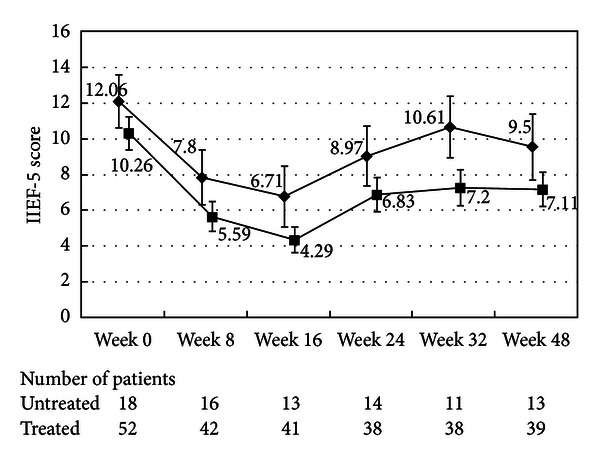
International Index of Erectile Function-5 score. The scores in the patients treated with an *α*1-blocker or anticholinergic agent (■) and the untreated patients (◆) are shown. The values are LS mean ± SE. *P* = 0.6575 (repeated ANOVA).

**Figure 6:**
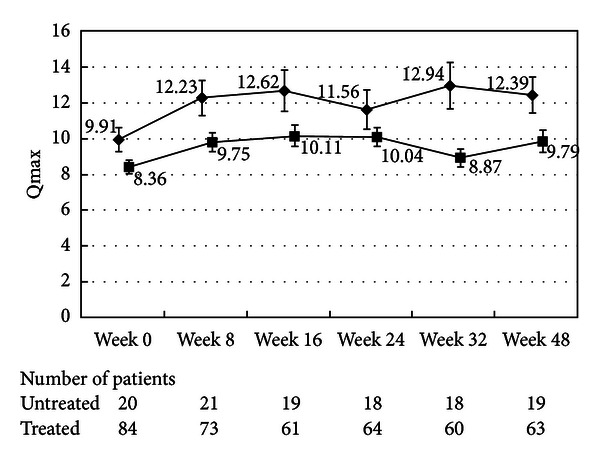
Maximal urinary flow rate. The scores in the patients treated with an *α*1-blocker or anticholinergic agent (■) and the untreated patients (◆) are shown. The values are LS mean ± SE. *P* = 0.1170 (repeated ANOVA).

**Figure 7:**
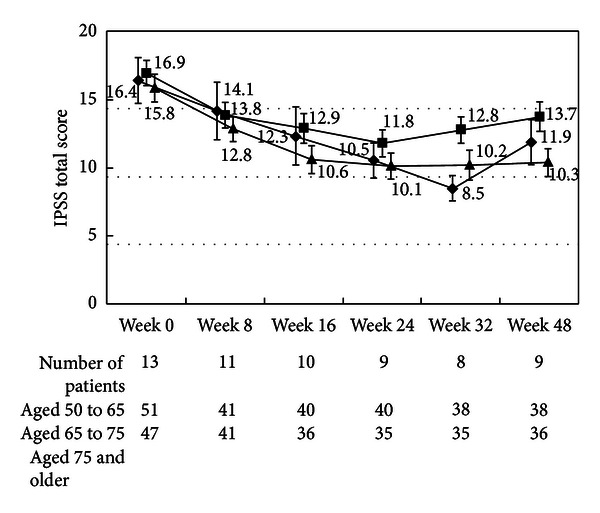
International Prostate Symptom Score total score by age. The scores in the patients aged from 50 to 65 (◆), the patients aged from 65 to 75 (■), and the patients aged 75 and older (▲) are shown. The values are LS mean ± SE. *P* = 0.2055 (repeated ANOVA).

**Figure 8:**
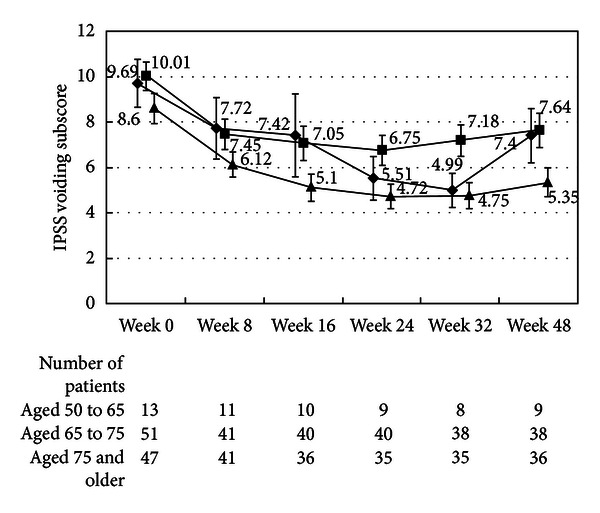
International Prostate Symptom Score voiding subscore by age. The scores in the patients aged from 50 to 65 (◆), the patients aged from 65 to 75 (■), and the patients aged 75 and older (▲) are shown. The values are LS mean ± SE. *P* = 0.7578 (repeated ANOVA).

**Figure 9:**
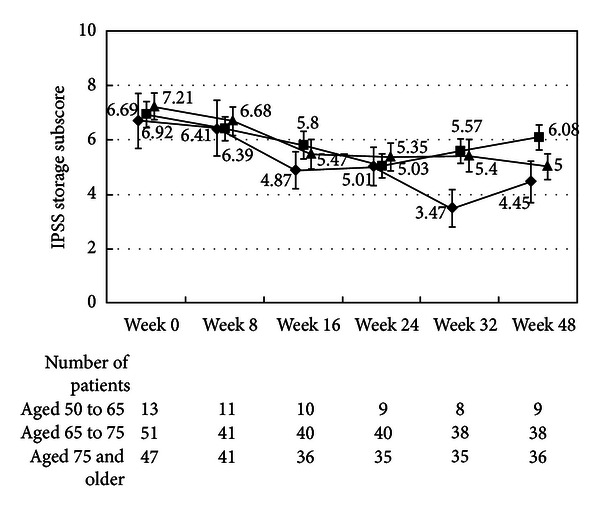
International Prostate Symptom Score storage subscore by age. The scores in the patients aged from 50 to 65 (◆), the patients aged from 65 to 75 (■), and the patients aged 75 and older (▲) are shown. The values are LS mean ± SE. *P* = 0.0290 (repeated ANOVA).

**Figure 10:**
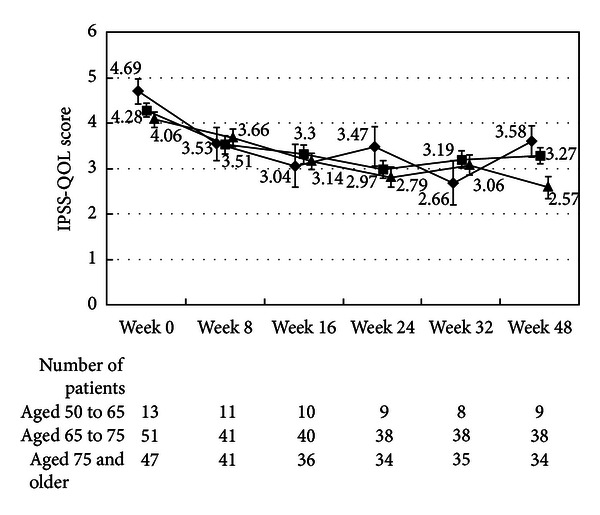
International Prostate Symptom Score quality of life score. The scores in the patients aged 50 to 65 (◆), the patients aged from 65 to 75 (■), and the patients aged from 75 and older (▲) are shown. The values are LS mean ± SE. *P* = 0.0001 (repeated ANOVA).

**Figure 11:**
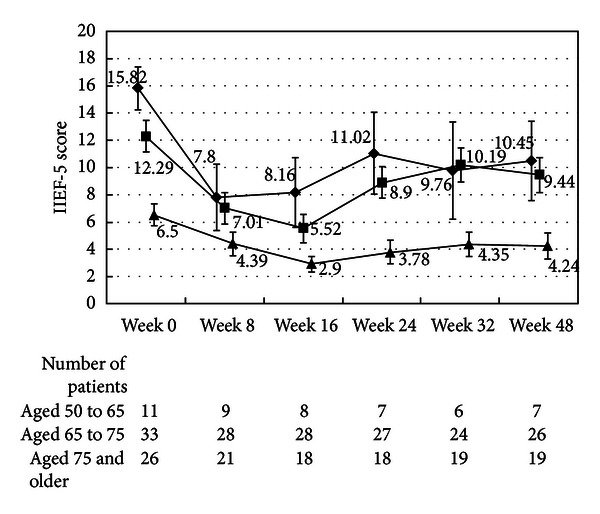
International Index of Erectile Function-5 score. The scores in the patients aged from 50 to 65 (◆), the patients aged from 65 to 75 (■), and the patients aged 75 and older (▲) are shown. The values are LS mean ± SE. *P* = 0.0093 (repeated ANOVA).

The changes in the IIEF-5 score are shown in Figures 5 and 11. Deterioration of erectile function, which was indicated by a decrease in the IIEF-5 score, was significant at week 8 and greatest at Week 16. The changes in IIEF-5 scores were significantly smaller in the patients aged 75 years or older than in the younger patients. The patients aged from 75 to 80 years continued to show a significant decrease in the IIEF-5 score at Week 8 through Week 32 after the cessation of CMA. The younger patients showed recovery of IIEF-5 score at Week 8 after cessation of CMA. 

## 4. Discussion

Our patients presented with a relatively high mean age, a large mean prostate volume, and a high mean PSA level when compared with the subjects of similar previous studies. Although nearly 80% of the patients in this study were treated with an *α*1-blocker or an anticholinergic drug, significant improvements from baseline values were observed in the total IPSS during CMA treatment and after cessation of the treatment. Each IPSS voiding and storage symptom subscore, except the nocturia subscore, improved during CMA treatment. Therefore, the continued improvements in the IPSS-QOL scores after cessation of the treatment indicated that CMA had lasting effects against LUTS and adequately satisfied the patients. In particular, the amelioration of voiding symptoms was durable in old patients aged 75 or more, whereas the amelioration of storage symptoms was sharp in young patients aged from 50 to 65 years. On the other hand, the Qmax increased by approximately 2 mL/s after 16 weeks of CMA treatment; the level of the increase was almost the same as that reported during dutasteride treatment for patients without *α*1-blocker pretreatment [5]. In a Japanese clinical trial of dutasteride, it took about 30 weeks for *Q*_max⁡_ to increase by at least 2 mL/s [6]. These results suggest that CMA's effects on urinary flow rate are similar in size to 5-ARIs' but have a faster onset. 

Previous studies by Ueki et al. and Ohtani et al. reported superior clinical efficacy of combined treatment with an *α*1-blocker and CMA compared with monotherapy with either of the 2, with respect to IPSS [7, 8]. In a randomized controlled study investigating the efficacy of the 48-month combination therapy with tamsulosin and dutasteride, combination therapy was superior to either monotherapy [9]. In the dutasteride monotherapy group, IPSS and *Q*_max⁡_ steadily improved during treatment, albeit at a slower rate than in the tamsulosin monotherapy group. However, in the tamsulosin monotherapy group, they reached the maximum at 3 months and decreased steadily thereafter. In the combination therapy group, the IPSS and *Q*_max⁡_ improved as early as 3 months to levels that were similar to those observed in the tamsulosin monotherapy group and continued to improve slowly until the end of the observation period. Similar results are reasonably expected with CMA used in place of 5-ARI. In this study, the IPSS and IPSS-QOL improvements were comparable between the patient subgroup being treated with concomitant *α*1-blockers and the untreated patient subgroup; the only significant difference was the greater improvement in *Q*_max⁡_ that was observed in the untreated patients. In this study, the prostate volume was reduced by 25% after the 16-week treatment with CMA. CMA has been shown to reduce prostate volume more rapidly and strongly than other antiandrogens and 5-ARIs [6, 10–12]; the reduction in the prostate volume ranged from 23 to 36% in patients receiving CMA for 16 weeks [10–12] compared to the 17.1% reduction that was found with a 16-week finasteride treatment [11] and the 4.8% reduction that was found with a 16-week allylestrenol treatment [10]. Moreover, the reduction rate of prostate volume after 52 weeks of long-term CMA treatment was 44%, [12] which was higher than the 24% or 34% reduction after 52 weeks of dutasteride treatment [6, 13]. Hepatic function disorder is uncommon with CMA when it is used at the approved dose (50 mg/day) for BPH. Other adverse reactions to CMA include sexual dysfunction, urinary frequency, and gastrointestinal disorders, which are similar in variety and incidence rates to the reactions to other androgen deprivation therapies. Erectile dysfunction was indicated by the decrease in IIEF-5, which was significant at Week 8, and greatest at Week 16 in this study. While patients aged 75 years or older constituted a significant portion (41%) of the patient population, the initial IIEF-5 and decrease in IIEF-5 was smaller in this subgroup compared to the younger patients. Therefore, for this high-age subgroup, erectile dysfunction may need to be individually treated only in patients who wish to preserve sexual function. 

Owing to the reduction in testosterone levels by CMA treatment, the incidences of sexual disability and erectile dysfunction were usually high in CMA-treated patients [11]. The incidence of erectile dysfunction was 2.3% in 3607 patients treated with CMA in a postmarketing surveillance whereas it was 3.2% in 403 patients treated with dutasteride in Japanese clinical trials. Although CMA is a progesterone derivative, gynecomastia is infrequent in patients treated with CMA in contrast to its frequency in patients treated with dutasteride. The incidence of gynecomastia was reportedly 0.19% in the postmarketing surveillance of CMA whereas it was 1.5% in Japanese clinical trials of dutasteride. The difference may be explained by the decreased estrogen levels in CMA-treated patients as opposed to the increased estrogen levels in patients treated with dutasteride or a nonsteroidal antiandrogen bicalutamide; they increase the level of testosterone, which is converted to estrogen by aromatases. Although more patients who are treated with combination therapy with dutasteride and tamsulosin reportedly experienced ejaculation disorder than those receiving *α*1-blocker monotherapy [14], no patient experienced ejaculation disorder in this study.

Since a low serum testosterone level induces metabolic syndrome and increases visceral fat due to a poor response to insulin and a low basal metabolic rate [15], patients treated with CMA for a long-term need a periodic checkup of metabolic syndrome. On the other hand, osteoporosis may not need to be concerned as an adverse reaction to CMA or 5-ARIs. Although there is no available data on CMA, a derivative of progesterone, progesterone generally induces differentiation of osteoblast and promotes bone formation. Meanwhile estrogen converted from serum testosterone that increases with 5-ARIs administration is speculated to induce Fas ligand-related apoptosis of osteoclast and inhibit bone resorption. The relationship between serum testosterone level and osteoporosis is controversial and has not been clarified, and a significant serum cutoff level of testosterone for osteoporosis has not been determined [16]. Some literatures have reported that testosterone at a level of >30 ng/mL would not cause osteoporosis [17–19].

Ever since its approval 30 years ago, the adverse effects of CMA on hepatic and sexual function have limited its use in general to elderly patients without hepatic disorder. It has been often used for short-term treatment preceding TURP because it sharply reduces the prostate volume and is useful for minimizing the risk of bleeding during the procedure. However, no large clinical study has tested the clinical effects of CMA on LUTS. Meanwhile, recent large clinical studies have demonstrated the efficacy of dutasteride and helped its introduction into the worldwide market. In that connection, there is no difference in the drug cost in long-term treatment between CMA and dutasteride in Japan. 

This study showed that CMA rapidly and effectively reduced the adenoma size, thereby improving LUTS. There were few severe adverse events to CMA, except for sexual dysfunction. Thus, CMA seems to be safe and especially beneficial for elderly patients with moderate-to-severe LUTS associated with BPH of a large prostate volume.

## Figures and Tables

**Table 1:** Baseline characteristics of the patients.

Characteristic	Classification	Number of patients (%) N = 114
	No	23 (20.2)
Pretreatment drug	Yes	91 (79.8)
	*α*1-blocker	91 (79.8)
	Anticholinergic agent	9 (7.9)

	50 to 65	14 (12.3)
	65 to 70	14 (12.3)
	70 to 75	39 (34.2)
Age (yrs)	75 to 80	27 (23.7)
	≥80	20 (17.5)
	Mean ± SD	**73.0 ± 6.8**
	Median	**73.0**
	Range (Min–Max)	**57.0–89.0**

	≤1.0	7 (6.5)
	>1.0 and ≤2.0	12 (11.2)
PSA at Week 0^†^ (ng/mL)	>2.0 and ≤4.0	31 (29.0)
	>4.0 and ≤6.0	22 (20.6)
	>6.0 and ≤10.0	31 (29.0)
	>10.0	4 (3.7)
	Mean	**3.66**

	20 to 30	22 (20.2)
	30 to 40	18 (16.5)
Prostate volume (mL)	40 to 55	31 (28.4)
	55 to 80	29 (26.6)
	≥80	9 (8.3)
	Mean	**46.15**

PSA: prostate-specific antigen; ^†^The day of the first dose.

**Table:** (a)

	Voiding scores														
	Incomplete emptying			Intermittency			Weak stream			Straining			Voiding subscore		
	*N*	Mean	SE	*N*	Mean	SE	*N*	Mean	SE	*N*	Mean	SE	*N*	Mean	SE
Week 0^†^	111	2.23	0.16	111	2.10	0.16	111	3.43	0.14	110	1.65	0.15	111	9.38	0.43
Week 8	93	1.56*	0.15	93	1.63*	0.15	93	2.52*	0.16	92	1.20*	0.14	93	6.92*	0.42
Week 16	86	1.32*	0.14	86	1.53*	0.17	86	2.27*	0.17	86	1.14*	0.14	86	6.27*	0.49
Week 24^‡^	84	1.24*	0.13	84	1.42*	0.14	84	2.11*	0.16	84	0.99*	0.13	84	5.76*	0.41
Week 32^§^	81	1.37*	0.15	81	1.35*	0.14	81	2.07*	0.17	79	1.19*	0.15	81	5.92*	0.42
Week 48^#^	83	1.42*	0.13	83	1.66*	0.17	83	2.30*	0.16	82	1.28	0.15	83	6.63*	0.47

**Table:** (b)

	Storage scores												IPSS total score			IPSS-QOL score		
	Frequency			Urgency			Nocturia			Storage subscore								
	*N*	Mean	SE	*N*	Mean	SE	*N*	Mean	SE	*N*	Mean	SE	*N*	Mean	SE	*N*	Mean	SE
Week 0^†^	110	2.61	0.15	110	2.11	0.16	111	2.34	0.12	111	7.02	0.33	111	16.40	0.64	111	4.24	0.11
Week 8	93	2.24*	0.16	92	1.86	0.16	93	2.45	0.12	93	6.52	0.33	93	13.45*	0.63	93	3.58*	0.13
Week 16	86	1.73*	0.15	86	1.35*	0.15	86	2.45	0.12	86	5.56*	0.34	86	11.83*	0.72	86	3.20*	0.13
Week 24^‡^	84	1.65*	0.13	84	1.43*	0.14	84	2.06*	0.11	84	5.15*	0.31	84	10.91*	0.63	81	2.95*	0.14
Week 32^§^	81	1.72*	0.14	81	1.39*	0.15	81	2.15	0.13	81	5.28*	0.34	81	11.21*	0.67	81	3.09*	0.14
Week 48^#^	83	1.92*	0.15	81	1.40*	0.14	83	2.11	0.12	83	5.43*	0.31	83	12.06*	0.7	81	3.01*	0.15

Dunnett's multiple comparison test; *P < 0.05; IPSS: International Prostate Symptom Score; QOL: quality of life; ^†^The day of the first dose; ^‡^Week 8 of follow-up period; ^§^Week 16 of follow-up period; ^#^Week 32 of follow-up period.

## References

[1] Oelke M, Bachmann A, Descazeaud A (2012). *Guidelines on the Management of Male Lower Urinary Tract Symptoms (LUTS), Including Benign Prostatic Obstruction (BPO)*.

[2] Crawford ED, Wilson SS, McConnell JD (2006). Baseline factors as predictors of clinical progression of benign prostatic hyperplasia in men treated with placebo. *Journal of Urology*.

[3] Shibata Y, Fukabori Y, Ito K, Suzuki K, Yamanaka H (2001). Comparison of histological compositions and apoptosis in canine spontaneous benign prostatic hyperplasia treated with androgen suppressive agents chlormadinone acetate and finasteride. *Journal of Urology*.

[4] Fujimoto K, Hirao Y, Ohashi Y (2011). Changes in serum prostate specific antigen and testosterone levels after chlormadinone acetate treatment in patients with benign prostatic hyperplasia: a prospective multicenter clinical study. *Hinyokika Kiyo*.

[5] Roehrborn CG, Nickel JC, Andriole GL (2011). Dutasteride improves outcomes of benign prostatic hyperplasia when evaluated for prostate cancer risk reduction: secondary analysis of the Reduction by Dutasteride of prostate Cancer Events (REDUCE) trial. *Urology*.

[6] Tsukamoto T, Endo Y, Narita M (2009). Efficacy and safety of dutasteride in Japanese men with benign prostatic hyperplasia. *International Journal of Urology*.

[7] Ueki O, Kawaguchi K, Katsumi T (1998). Clinical efficacy and reduction effect on prostatic volume of chlormadinone acetate combined with tamsulosin hydrochloride in benign prostatic hyperplasia patients insufficiently treated with tamsulosin hydrochloride only. *Hinyokika Kiyo*.

[8] Ohtani M, Kikuchi K, Tsuchiya A (2000). A randomized long-term comparative study of clinical efficacy of *α*1-blocker with or without antiandrogen therapy for benign prostatic hyperplasia: focusing on improvement of I-PSS. *Hinyokika Kiyo*.

[9] Roehrborn CG, Siami P, Barkin J (2009). The influence of baseline parameters on changes in international prostate symptom score with dutasteride, tamsulosin, and combination therapy among men with symptomatic benign prostatic hyperplasia and an enlarged prostate: 2-year data from the CombAT study. *European Urology*.

[10] Shida K, Koyanagi T, Kawakura K (1986). Clinical effects of allylestrenol on benign prostatic hypertrophy by double-blind method. *Hinyokika Kiyo*.

[11] Aso Y, Homma Y, Kumamoto Y (1995). Phase III study of 5 alpha-reductase inhibitor, MK-906 in patients with benign prostatic hyperplasia. A comparative double blind study with chlormadinone acetate long acting tablets. *Hinyouki Geka*.

[12] Kogawa T, Yanagiya H, Takashima T (1993). Clinical evaluation of the long-term treatment with chlormadinone acetate on patients with benign prostatic hypertrophy. *Hinyokika Kiyo*.

[13] Roehrborn CG, Boyle P, Nickel JC, Hoefner K, Andriole G (2002). Efficacy and safety of a dual inhibitor of 5-alpha-reductase types 1 and 2 (dutasteride) in men with benign prostatic hyperplasia. *Urology*.

[14] Roehrborn CG, Siami P, Barkin J (2010). The effects of combination therapy with dutasteride and tamsulosin on clinical outcomes in men with symptomatic benign prostatic hyperplasia: 4-year results from the CombAT study. *European Urology*.

[15] Muller M, Grobbee DE, den Tonkelaar I, Lamberts SWJ, van der Schouw YT (2005). Endogenous sex hormones and metabolic syndrome in aging men. *Journal of Clinical Endocrinology and Metabolism*.

[16] Fink HA, Ewing SK, Ensrud KE (2006). Association of testosterone and estradiol deficiency with osteoporosis and rapid bone loss in older men. *Journal of Clinical Endocrinology and Metabolism*.

[17] Stoch SA, Parker RA, Chen L (2001). Bone loss in men with prostate cancer treated with gonadotropin-releasing hormone agonists. *Journal of Clinical Endocrinology and Metabolism*.

[18] Preston DM, Torréns JI, Harding P, Howard RS, Duncan WE, Mcleod DG (2002). Androgen deprivation in men with prostate cancer is associated with an increased rate of bone loss. *Prostate Cancer and Prostatic Diseases*.

[19] Wang W, Yuasa T, Tsuchiya N (2008). Bone mineral density in Japanese prostate cancer patients under androgen-deprivation therapy. *Endocrine-Related Cancer*.

